# Trends in extracellular matrix biology

**DOI:** 10.1007/s11033-022-07931-y

**Published:** 2022-11-07

**Authors:** Konstantina Kyriakopoulou, Zoi Piperigkou, Kyriaki Tzaferi, Nikos K. Karamanos

**Affiliations:** 1 Biochemical Analysis & Matrix Pathobiology Research Group, Laboratory of Biochemistry, Department of Chemistry, University of Patras, 265 04 Patras, Greece; 2 Foundation for Research and Technology-Hellas (FORTH), Institute of Chemical Engineering Sciences (ICE-HT), 261 10 Patras, Greece

**Keywords:** Extracellular matrix, Interaction databases, Tissue integrity, Cell signaling, Functional properties, Disease, ECM targeting

## Abstract

Extracellular matrixes (ECMs) are intricate 3-dimensional macromolecular networks of unique architectures with regulatory roles in cell morphology and functionality. As a dynamic native biomaterial, ECM undergoes constant but tightly controlled remodeling that is crucial for the maintenance of normal cellular behavior. Under pathological conditions like cancer, ECM remodeling ceases to be subjected to control resulting in disease initiation and progression. ECM is comprised of a staggering number of molecules that interact not only with one another, but also with neighboring cells via cell surface receptors. Such interactions, too many to tally, are of paramount importance for the identification of novel disease biomarkers and more personalized therapeutic intervention. Recent advances in big data analytics have allowed the development of online databases where researchers can take advantage of a stochastic evaluation of all the possible interactions and narrow them down to only those of interest for their study, respectively. This novel approach addresses the limitations that currently exist in studies, expands our understanding on ECM interactions, and has the potential to advance the development of targeted therapies. In this article we present the current trends in ECM biology research and highlight its importance in tissue integrity, the main interaction networks, ECM-mediated cell functional properties and issues related to pharmacological targeting.

## ECMs: dynamic regulatory networks in tissue remodeling and integrity

Human tissues are mainly constituted of cells including fibroblasts, immune, endothelial, and epithelial cells, and various types of non-cellular ECM networks. The composition of ECMs differs between tissues, developmental stages, and pathophysiological conditions. ECM macromolecular networks orchestrate cellular properties through signaling cascades, exhibiting paramount importance in cell communication that guides cellular behavior in normal homeostasis and disease conditions [1, 2].

**Fig. 1:**
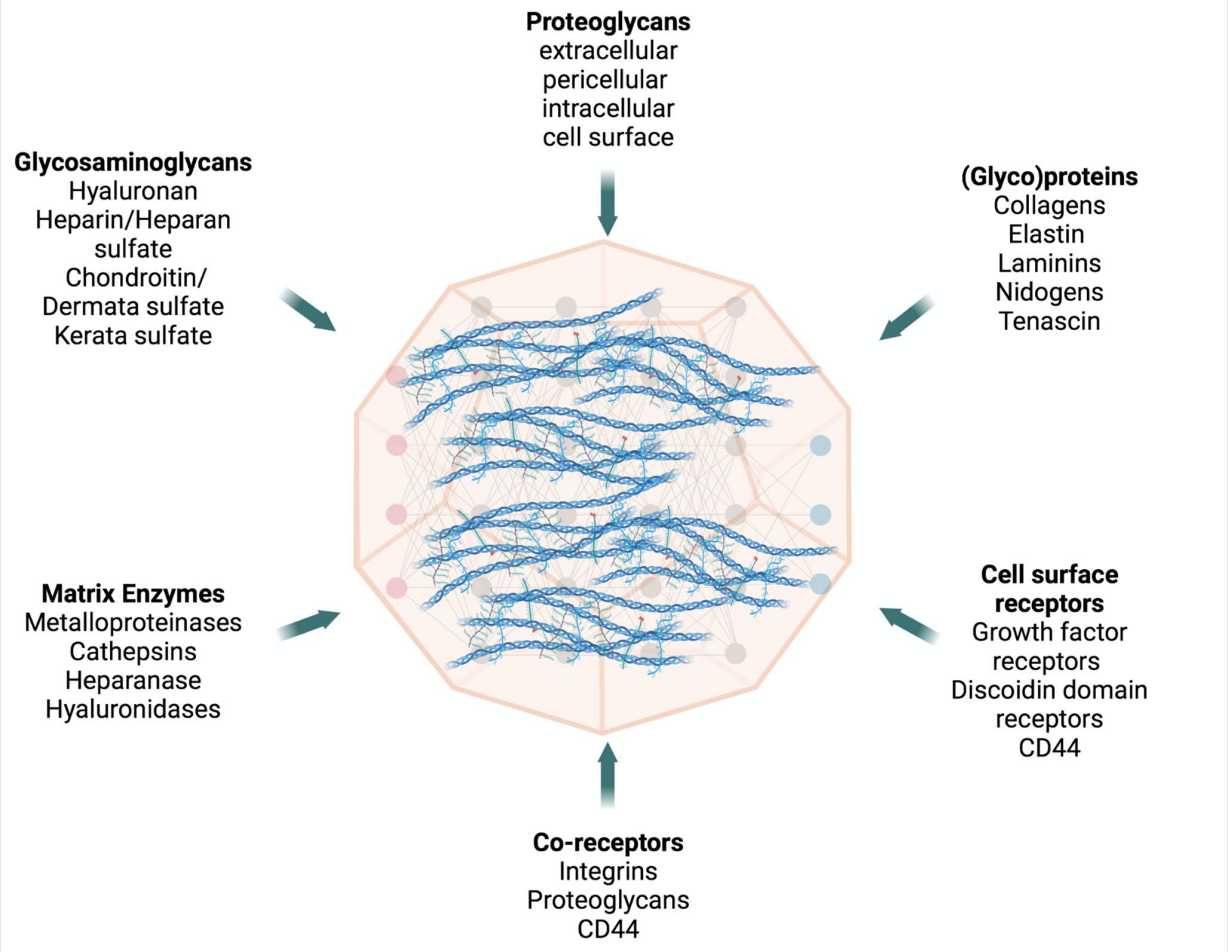
Major macromolecular components of the 3D matrix network. The matrix biomolecules that contribute to the structural and functional stability and multitasking processes in cells and tissues include glycosaminoglycans, proteoglycans, growth factors and their receptors, proteolytic and non-proteolytic enzymes. *Created with Biorender.com*

The multitasking ECM is formed by hundreds of different building blocks, interacting macromolecules and bioactive modulators that upon cell-matrix communication affect cell phenotype and functions [3]. The core of ECM network is consisted of structural and functional macromolecules, such as proteoglycans and glycosaminoglycans (PGs/GAGs), collagens, elastin, laminins, tenascins, nidogens as well as cell surface receptors and co-receptors, including integrins and hyaluronan (HA) receptor, CD44. Matrix remodeling is finely tuned by the enzymatic actions of matrix-degrading enzymes, as proteases, including matrix metalloproteinases (MMPs), adamalysins and glycosidases, such as heparanase and hyaluronidases (Fig. 1) [4, 5].

The content and structural features of matrix components segregate ECMs into interstitial and pericellular ones, the latter being the basement membrane (ΒΜ). Interstitial matrices mainly consist of fibrillar collagens, fibronectin, PGs and matricellular proteins. The main ECM components of the ΒΜ consist of collagen IV, laminins, nidogens, and the heparan sulfate PGs, perlecan and agrin [3]. It is worth noticing that the formulation of ECMs may constantly be adapted depending on mechanical or biochemical signals, resulting in a fine-tuned ECM remodeling procedure. Matrix macromolecules are finely orchestrated to form a 3D dynamic ECM meshwork being the most important and abundant native biomaterial in human organisms.

ECMs surround cells and act as their physical barrier, while also being constituents of tissues along with a variety of cells, such as endothelial and epithelial cells, fibroblasts, pericytes and immune system cells. Interestingly, in tissues, ECMs significantly contribute to the structural support of the parenchymal cells [6]. The biological functions of ECMs differ depending on the type of ECM and the tissue. Interstitial matrices and BMs exhibit certain similarities, though at the same time their functionality is distinct. Specifically, interstitial connective tissue matrix contributes to the organization of space between cells and regulates cells and tissues interactions, while BM acts on tissue integrity, viscoelasticity and biomechanical signaling, as well as to modulate cell-cell interactions [1]. In tissues, ECMs present an array of functions that vary from tissue barrier, growth and shaping to cell adhesion, migration and signaling [7, 8]. Of note, during brain development, the parenchymal and endothelial BMs are crucial for the formation of the blood-brain barrier. In the skin, BM is the underlying support on which the basal epithelial cells accumulate and give rise to the stratified skin layers [9–11]. In general, the tissue matrix constantly remodels through a delicately balanced circle of synthesis and degradation, to maintain tissue homeostasis and normal functionality.

## ECMs as 3D complex interacting functional networks

The functional interactions of matrix bioactive effectors with the conterminous microenvironment are key players in regulating tissue homeostasis and pathological conditions, including cancer [3]. Interactions within the ECM are quite complex and responsible for generating signals to remodel vital cell properties, such as proliferation, migration, adhesion, and differentiation [12]. For instance, the extracellular PG, decorin, interacts with epidermal growth factor receptor (EGFR), Met and vascular endothelial growth factor receptor 2 and regulates the assembly of collagen fibrils [13, 14]. Moreover, lumican interacts with MMP14, integrins and collagen type I [15], while the intracellular PG, serglycin interacts with chemokines, zymogens and MMPs, as pro-MMP9 and MMP13 [16]. The cell-surface PG, syndecan-1 (SDC-1) dynamically interacts with α6β4, ανβ3, ανβ5 integrins to regulate angiogenesis, cell invasion and survival, whereas SDC-4 interacts with EGFR, α6β4 and α5β1 integrins to promote wound healing and focal adhesion [17].

A thorough understanding of these interactions may benefit the matrix-centric tissue engineering to systematically regulate cellular functions in respect to human pathologies.

### ECM interacting networks databases – the matrix interactome code

Protein-protein interactions among matrix components are particularly important for the complex web of functional associations between biomolecules that mediates cell behavior in normal homeostasis and disease conditions [8]. Biomolecular networks allow the inference of specific cell properties through functional association of matrix components, and support drug target discovery, therefore they are widely used in modern drug design and pharmacology [18, 19]. Several online resources dedicated to organism-wide protein association networks have already developed using curated proteomic data on the ECM of normal and diseased tissues (Table 1).

**Table 1:** Available databases predicting interaction networks among functional matrix components

Database	Hyperlink	Reference
Adhesome	http://www.adhesome.org	[20]
Cytoscape	https://cytoscape.org	[21]
DAVID	https://david.ncifcrf.gov	[22]
FunCoup	http://funcoup.sbc.su.se	[23]
FunRich	http://www.funrich.org	[24]
IID	http://ophid.utoronto.ca/ophidv2.204/	[25]
IMP 2.0	http://imp.princeton.edu	[26]
MatrisomeDB	http://matrisomedb.pepchem.org	[27]
Matrix DB	http://matrixdb.univ-lyon1.fr	[28]
STRING	https://string-db.org	[29]

The adhesive interactions of cells with their environment through the integrin family of transmembrane receptors may be predicted using Adhesome [20]. Functional enrichment and interaction network analysis of genes and proteins may be integrated using searchable resources as demonstrated in Table 1 [i.e., Cytoscape [21], DAVID [22], FunCoup [23], FunRich [24], IID [25], IMP 2.0 [26], MatrisomeDB [27], MatrixDB [28], STRING [29]].

The Adhesome network is a literature-based protein-protein interaction network that was developed from the biomedical literature. The network is made of known interactions and cellular components constituting the focal adhesion complex in mammalian cells [20]. MatrisomeDB is a searchable database that integrates experimental proteomic data on the matrix composition in normal and diseased tissues [30]. It also provides live cross-referencing to gene and protein databases for every matrix and matrix-associated gene [27]. Matrix DB reports interactions established by matrix proteins, PGs and polysaccharides with individual polypeptide chains or with multimers (i.e., collagens, laminins, thrombospondins) [31]. Moreover, it stores experimental data established by full-length proteins, matricryptins, GAGs, lipids and cations [28]. Last but not least, the STRING resource aims to integrate known protein-protein physical interaction networks and functional associations by functional enrichment analysis containing more than 14 000 organisms [29].

## ECM-mediated cell signaling and functional properties

**Fig. 2:**
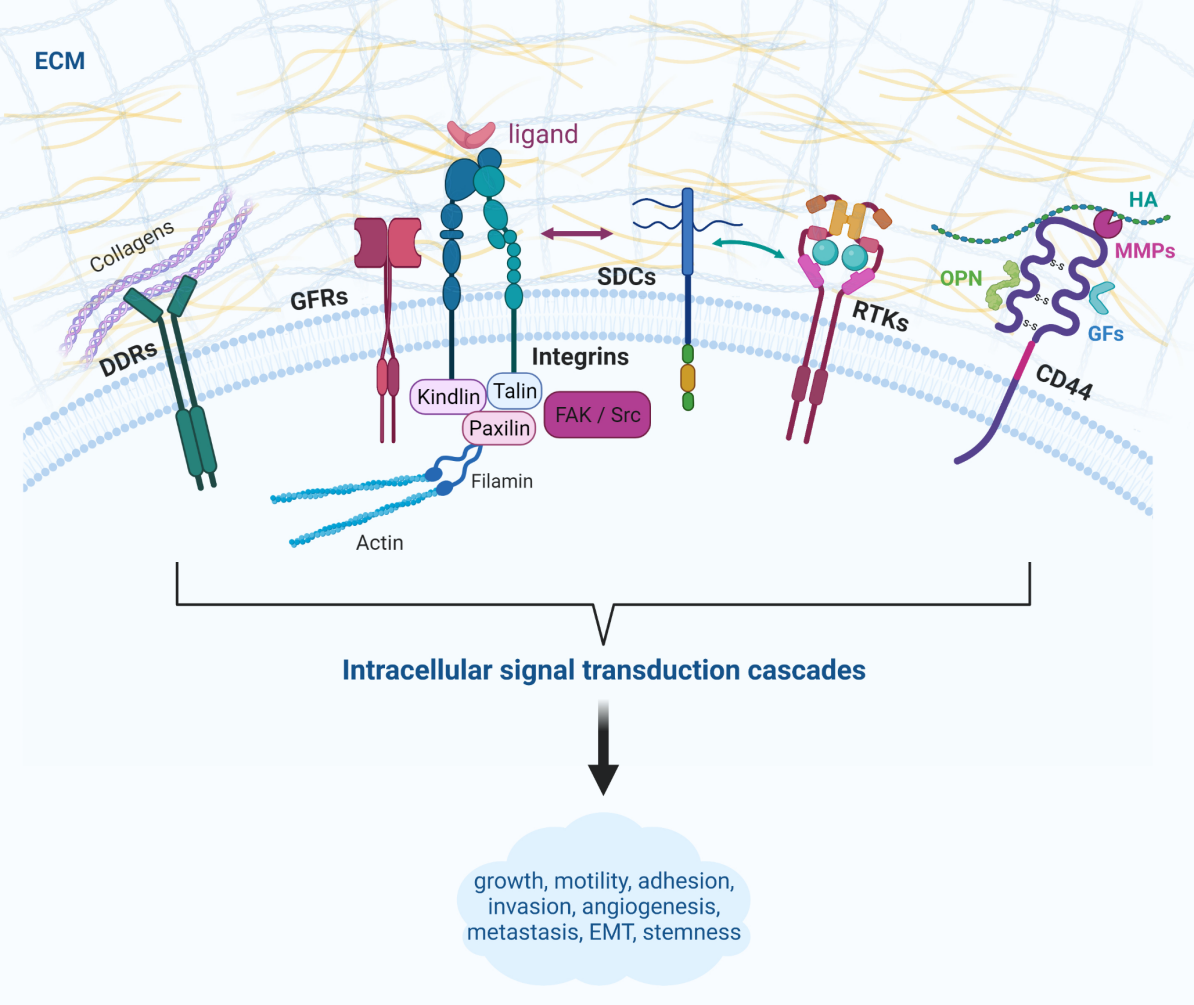
ECM-mediated outside-in signal transduction. Cell-surface receptors act as liaisons between ECM effectors and intracellular signaling cascades. Particularly, integrins, key anchorage proteins, act also as signal transducers after their interaction with cytoskeleton-associated molecules (i.e., talin, vinculin, paxillin), that leads to the activation of focal adhesion kinase (FAK) and Src-family protein tyrosine kinases (SFKs) and subsequently modulation of downstream effectors like PI3K/Akt, ERK and Rho GTPases. Integrins usually co-localize beside GFRs at focal adhesions and can also signal interdependently with SDCs. SDCs additionally function as co-receptors for GFRs. Furthermore, DDRs are a unique sub-family of RTKs that do not bind integrin but respond to collagens as ligands. Finally, CD44 acts as a receptor for various ECM molecules (i.e., HA, OPN, MMPs) to prompt activation of downstream signaling, while functioning as co-receptor for GFs, cytokines and other ECM components to promote angiogenesis, EMT and stemness. *Created with Biorender.com*

Cells continuously audit the biochemical composition of the surrounding matrix utilizing various cell surface receptors including integrins, discoidin domain receptors (DDRs), SDCs and CD44, to fine-tune intracellular signaling pathways respectively [3]. Integrins, transmembrane heterodimers of α and β subunits, can bind to various proteins that contain an RGD (arginine–glycine–aspartic acid) domain (i.e., collagens, fibronectin etc.) and act not only as anchorage proteins, but also as signal transducers to intracellular molecules to control cell behavior [32]. Particularly, integrins cluster with actin and create complexes that reinforce focal adhesion interactions and commence the assembly of adhesomes. Likewise, following growth factor stimulation, they co-localize at focal adhesions alongside growth factor receptors (GFRs), signaling molecules, (i.e., FAK, Src) and cytoskeleton-associated molecules like vinculin, talin and paxillin (Fig. 2). This way, integrins ultimately modulate the activity of downstream effectors, such as PI3K/Akt, JNK, ERK and the Rho GTPases [33]. Additionally, integrins are able to intervene in the rate of growth factor receptor’s internalization and subsequent degradation [34]. DDRs are affiliated with the receptor tyrosine kinases (RTKs), though they present unique characteristics, especially their ability to bind collagens [3, 35].

SDCs, transmembrane proteoglycans with cell and tissue-specific expression patterns, are receptors mainly associated with adhesion [36]. What’s more, SDCs signal synergistically with integrins through clustering and serve as co-receptors of growth factor receptors (Fig. 2) [37]. CD44 is one of the cell adhesion molecules (CAMs) and is a complex glycoprotein that associates with ECM components namely HA, osteopontin (OPN) and MMPs, to stimulate downstream signaling pathways such as PI3K/Akt [38, 39] (Fig. 2). Nevertheless, CD44 also interacts with numerous other ECM molecules, growth factors and cytokines and advances tumor growth, angiogenesis, metastasis and cancer stem cell (CSC)-related properties [40].

ECM, as a highly dynamic macromolecular network of vital importance for cells, is well established that it determines and influences the morphology and fundamental cellular properties, including proliferation, migration, adhesion, polarity and angiogenesis [4]. The landscape by which the biology of ECM regulates cell functionality is complex. ECM effects on the cells can be differentially mediated either by the direct binding of cell surface receptors or co-receptors that modulates cell anchorage, mechanotransduction and intracellular signaling pathways, or by the remodeling due to aberrant presentation of growth factors and the actions of enzymes [41, 42]. The various ECM macromolecules implicated in main diseases are presented in Table 2. In pathological conditions like cancer, cells undergo significant changes in the molecular level that drive tumor progression and many researchers have focused on detailing these changes in the ECM. Elevated collagen deposition is often linked to more aggressive morphological characteristics and increased invasion, while the collagen fiber alignment is tightly connected to cell migration and tumor progression [43, 44]. Moreover, collagen rich ECMs with disproportionate cross-linking lead to stiffer microenvironments, which has been shown to promote invasion and metastasis through induction of epithelial-to-mesenchymal transition (EMT) and stemness [45–47]. On the other hand, fibronectin fibers are less stiff and more relaxed than collagen fibers, yet FN-enriched matrices tend to promote more malignant phenotype, since it is well-established that fibronectin is a key driver of EMT [48]. The different actions between collagen and fibronectin are usually attributed to distinct integrin dimer binding, which in turn activates alternative intracellular signaling pathways [49]. Furthermore, mutations or alterations in the expression of crucial ECM effectors like matrix remodeling enzymes (i.e., MMPs), heparan sulfate proteoglycans (HSPGs) and CD44 greatly influence disease progression. For example, higher MMPs expression and activity levels guides invasion and metastasis via, among others, promotion of invadopodia formation [48, 50]. In addition, SDCs govern angiogenesis and migration by acting as co-receptors of growth factor signals and have recently been recognized as biomarkers of stemness in breast cancer [17, 51, 52]. Finally, CD44 is also implicated as a marker for the induction of cancer stem cell (CSC) phenotype and thus, the therapeutic resistance in various cancers [53, 54].

**Table 2:** Extracellular matrix (ECM) macromolecules and association with main diseases

ECM molecule	Disease
**Collagen**	
collagen type I	fibrosis; pulmonary diseases; cancer
collagen type III	fibrosis; cancer
collagen type V	fibrosis
collagen type VI	pulmonary diseases
**Glycoproteins**	
elastin	fibrosis; pulmonary diseases
laminins	cancer
fibronectin	fibrosis; osteoarthritis; cancer
**GAGs**	
hyaluronan	fibrosis; inflammation; osteoarthritis; cancer
**Proteoglycans**	
versican	fibrosis; inflammation; cancer
aggrecan	osteoarthritis
brevican	cancer
perlecan	cancer
biglycan	fibrosis; osteoarthritis; cancer
decorin	osteoarthritis; cancer
lumican	myocardial fibrosis
fibromodulin	cancer
syndecans	fibrosis; cancer
glypicans	cancer
serglycin	cancer
**Integrins**	fibrosis; cancer

## The role of ECM in disease development and progression

Abnormal ECM remodeling is one of the leading causes of pathological conditions including cancer, fibrosis and osteoarthritis (Table 2). Particularly, the excessive ECM degradation causes tissue destruction, while the excessive synthesis and deposition of ECM, observed in chronic or acute tissue injuries, lead to fibrosis [55].

Fibrosis is a dynamic and reversible process, which is characterized as a heterogeneous disorder of connective tissue. ECM stiffness and disorganization because of non-canonical tissue repair, affect ECM signaling. The main components of fibrotic ECM are heterotypic fibrils of collagen type I, III and V, elastin, fibronectin, HA and versican aggregates, matricellular proteins and cross-linking enzymes like lysyl oxidase (LOX) [16, 56]. Generally, in pulmonary diseases, fragments of degraded collagen type I, VI and elastin are released to the circulation, where they induce eosinophil inflammation and development of emphysema [57]. Secreted ECM molecules in fibrous matrix interact with cell surface receptors including integrins. Integrins and SDCs can facilitate profibrotic signaling. In addition, it has been found that MMP3 and MMP7 are upregulated in lung fibrosis where induce the EMT program [16].

ECM remodeling promotes cardiac stiffness and therefore leads to heart failure. The deposition of collagen is a leading cause for the formation of atherosclerotic plaques and its degradation by MMPs is a risk factor for plaque rupture [58]. Among the most common ECM components involved in cardiac remodeling are extracellular and cell surface proteoglycans. It is worth mentioning that alterations in GAG chains increase PGs affinity for incoming into arterial wall low-density lipoproteins (LDLs), which are responsible for plaque progression [58]. Small leucine-rich proteoglycans (SLRPs) bind to collagen fibrils and regulate collagen organization, participating in cardiac fibrosis development. It is hypothesized that lumican induces myocardial fibrosis as it can control cellular expression and post-translational modifications of main cardiac remodeling molecules [59]. What is more, decorin regulates angiogenesis and thus is involved in the cardiac function recovery after injury [60].

Osteoarthritis is a musculoskeletal disease in which irreversible collapse of cartilage occurs. The accumulation of proteolytic enzymes and reactive oxygen species (ROS) in the injured tissue induces the degradation of collagen, aggrecan and other ECM components generating bioactive fragments of aggrecan, fibronectin and HA, which in turn proceed inflammation and catabolism [16, 61, 62]. Indeed, MMP3,MMP7 and MMP9 are overexpressed in the cartilage of patients with osteoarthritis [63]. Decorin and biglycan, two members of the SLRPs family, are associated with the initiation and progression of osteoarthritis, as both are upregulated. Notably, at the late stage of osteoarthritis, soluble fragments of biglycan are released to the synovial fluid and facilitate the loss of sulfated GAGs via the activation of NF-κΒ [64].

### ECM association with inflammatory responses and cancer

Tumor tissue is stiffer in comparison with the normal one and includes inflammatory mediators. This environment increases the expression of fibronectin-EDB, which promotes angiogenesis [65], whereas toll-like receptors (TLRs) induce proinflammatory signaling through binding to fibronectin and HA or cooperating with biglycan [16]. During inflammation, large amounts of ECM macromolecules including collagen type I, III and HA are produced inducing EMT and the dedifferentiation of epithelial cells into activated fibroblasts. Thus, epithelial cells acquire mesenchymal properties and move to distant sites. In addition, fibroblasts regulate the organization of collagen fibers. In case of desmoplastic stroma, collagen fibers are aligned in an ordered fashion, facilitating cancer cell migration [65]. Tumor progression is facilitated via the interaction between laminins and integrins. Furthermore, tumor cells and CAFs release MMPs, disintegrin and metalloproteinase domain-containing proteins (ADAMs), ADAMs with thrombospondin motifs (ADAMTSs), urokinase plasminogen activator (uPA) and cathepsins, which are involved in ECM molecules degradation [41]. Particularly, the plasminogen activation system is associated with tumor initiation and progression, while overexpression of MMP2, 3, 9, 13 and 14 augments cancer cell aggressiveness, stimulating EMT. Remarkably, MMP14 (MT1-MMP) has a very important role in cancer cell invasion and metastasis due to its regulatory role in invadopodia functions and its ability to degrade ECM molecules and basically collagen [41]. Versican regulates cell proliferation and metastasis by interacting with HA, TLRs and activating EGFR, or via ADAMTS-1-mediated proteolytic cleavage. On the other hand, proteolytic cleavage of brevican induces cancer cell adhesion and motility [16]. The interaction of decorin with various receptors in tumor niche exhibits antiproliferative and anti-angiogenic effects, whereas the interplay among biglycan and TLRs induces inflammation [66]. In glioblastoma cells, upregulated fibromodulin binds to collagen type I and promotes the activation of integrin-FAK-Src-Rho-ROCK signaling cascade causing tumor cell migration [67], while in lung cancer, fibromodulin promotes angiogenesis by increasing the expression of angiogenic factors [68]. Similarly, perlecan induces angiogenesis, cell proliferation, invasion, migration and drug resistance through the binding of heparan sulfate (HS) chains with growth factors, facilitating their presentation in cell surface receptors [16, 69]. In breast cancer cells, serglycin is upregulated and is correlated with increasing aggressiveness of tumor cells, as it promotes the expression of degrading enzymes, mesenchymal markers and the secretion of interleukin-8 (IL-8) [70]. The cell surface proteoglycans, SDCs and glypicans, are involved in tumor progression, acting either as tumor promoters or as tumor suppressors. Glypicans regulate growth factor signaling cascades, while SDCs, except for growth factors, interact with integrins regulating cancer cell functions [16]. Notably, SDC-1 is involved in exosomes biogenesis and regulates the exosome packaging [66].

## Pharmacological applications, diagnostics and ECM targeting

Collagen can be combined with several compounds such as GAGs to form scaffolds, which are used for the regeneration of cartilage, bone, tendon, burned skin, lung, and cornea. Furthermore, collagen-elastin scaffolds seem to be suitable for vascular tissue engineering, whereas collagen fragments have been used for wound healing [71]. Correspondingly, HA due to its structural properties, is used in regenerative medicine, generating scaffolds with great mechanical properties [72]. Notably, it is suggested that the molecular weight of HA affects its function in bone regeneration [73]. Finally, SDC-4 is recommended as a promising biomarker for tissue regeneration [74].

**Fig. 3:**
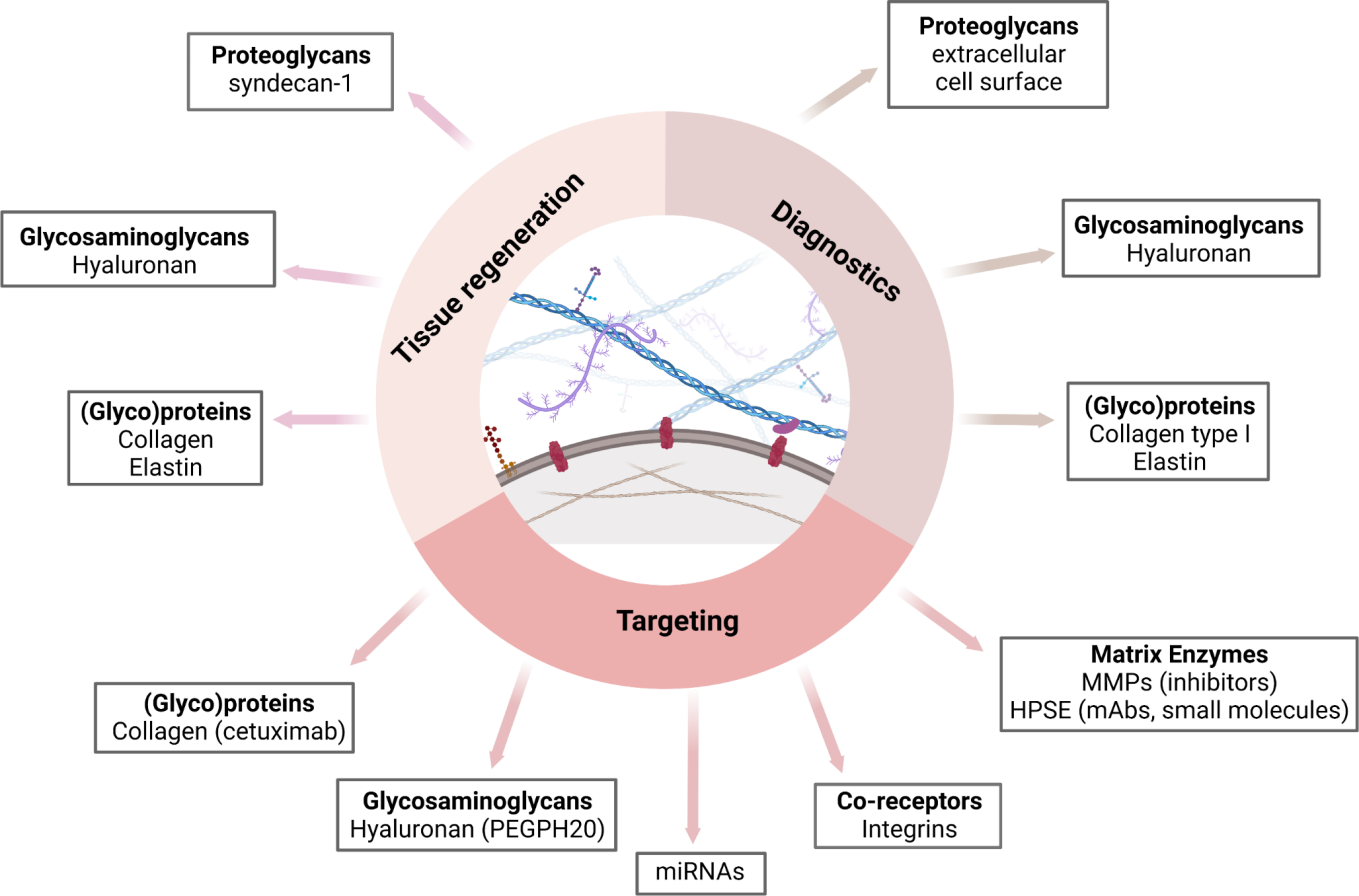
Pharmacological applications, diagnostic value and targeting of the main ECM components. Collagen, elastin and hyaluronan are important molecules for tissue regeneration and diagnosis of cancer and osteoarthritis. SDC-4, also, is used for tissue regeneration. Collagen can be targeted by monoclonal antibody, cetuximab, while hyaluronan is degraded by a recombinant human hyaluronidase, PEGPH20. The extracellular and cell surface proteoglycans are useful tools for cancer, osteoarthritis and heart disease diagnosis. Integrins are potential targets for the regulation of ECM remodeling in disease and matrix enzymes (MMPs, HPSE) are targeted by inhibitors or monoclonal antibodies respectively. An alternative strategy for ECM biosynthesis regulation are miRNAs. *Created with Biorender.com Abbreviations: HPSE; heparanase, mAbs; monoclonal antibodies, MMPs; metalloproteinases*

Plenty of ECM molecules are used as biomarkers in cancer prognosis and diagnosis (Fig. 3). Namely, high amounts of collagen type I fragment, ICTP, in serum of preoperated patients with triple negative breast cancer (TNBC) or luminal B subtypes may be a great biomarker for better patient prognosis [75]. On the other hand, N-telopeptide of collagen type I, appears to have high sensitivity and specificity and consequently can be used as a biomarker for bone metastasis in patients with lung cancer [76]. Moreover, increased elastin fragments produced by MMP7, 9 and 12, observed in the serum of lung cancer patients, are potential biomarkers for this type of cancer [77]. In osteoarthritis, degradation and synthesis products of collagen have been evaluated as predictive biomarkers of the development and progression of the disease. Further, other ECM components such as PGs, HA, aggrecan and glycoproteins seem to be useful tools for osteoarthritis diagnosis [78]. HA fragments possess different roles depending on their size and concentration. For instance, low molecular weight HA fragments exhibit an angiogenic effect and their presence in the serum of breast cancer patients is associated with lymph node metastasis [79]. Decorin is an anti-tumor factor, and its increased levels are associated with better survival and treatment response in cancer patients, including breast cancer. On the contrary, biglycan is a potential biomarker, whose overexpression relates to poor survival of colorectal, gastric and esophageal cancer patients [80]. Likewise, biglycan belongs to potential biomarkers of cardiac disease and contributes to the identification of patients, who may benefit from statin therapy [59]. The methylation of SDC-2 gene is a common event in precancerous lesions and its presence in bowel lavage fluid is useful for detecting patients with colorectal cancer [81]. Furthermore, shed PGs such as SDC-1 and SDC4 are promising blood biomarkers in heart disease [82]. Finally, it has been found that high glypican-1 is also a tumor marker of hepatocellular cancer [83].

Targeting of ECM molecules is a very important approach for therapeutic purpose against cancer and fibrosis. They have been developing four therapeutic strategies targeting collagen, so far. These comprise the inhibition of collagen synthesis, the degradation of stromal collagen, the suppression of collagen cross-linking by inhibiting LOX activity and the blocking of collagen interactions with integrins, which are extensively described in previous review article [65]. For example, collagen type I targeting of cetuximab, a monoclonal antibody against EGFR, has positive effects in epidermoid cancer therapy [11]. Respectively, there are three categories of therapeutic strategies targeting HA, the blocking of HA signaling, the inhibition of HA synthesis by the well-known inhibitor 4-MU and the degradation of HA [65]. An example of the last one category is the degradation of HA by PEGPH20, a recombinant human hyaluronidase, which leads to better survival of pancreatic ductal adenocarcinoma patients and improves drug delivery [11]. ECM biogenesis can also be regulated by miRNAs [84] and therapeutics based on miRNAs are candidates for clinical development [65, 85]. Another approach in order to control ECM homeostasis is the regulation of degrading enzymes like MMPs [86]. However, MMPs inhibitors have failed in clinical trials [11]. In contrast, heparanase inhibition by monoclonal antibodies, small molecule inhibitors and modified heparin effects, is considered to have anti-cancer potential [59]. Integrins are promising targets against inflammatory bowel disease and in multiple sclerosis as well as in the prevention of thrombotic complications [11]. Finally, proteoglycans, in particular aggrecan, mimicking polymers have shown satisfactory results in osteoarthritis clinical applications [74].

## Concluding remarks

ECM is a 3D complex network, which can be used as a natural biomaterial. This dynamic network regulates tissue organization and homeostasis. ECM macromolecules interact with cells and surface receptors, affecting cell signaling and therefore determine cell morphology and functions. However, abnormal ECM remodeling leads to the development and progression of several diseases. Thus, many ECM molecules have been evaluated as therapeutic targets whereas many of them are promising markers for disease prognosis and diagnosis.

## References

[1] Theocharis AD, Skandalis SS, Gialeli C, Karamanos NK (2016). Extracellular matrix structure. Adv Drug Deliv Rev.

[2] Karamanos NK, Piperigkou Z, Passi A (2021). Extracellular matrix-based cancer targeting. Trends Mol Med.

[3] Manou D, Caon I, Bouris P et al (2019) The Complex Interplay Between Extracellular Matrix and Cells in Tissues. pp 1–20 10.1007/978-1-4939-9133-4_1 30825161

[4] Iozzo RV, Theocharis AD, Neill T, Karamanos NK (2020). Complexity of matrix phenotypes. Matrix Biol Plus.

[5] Karamanos NK, Theocharis AD, Neill T, Iozzo RV (2019). Matrix modeling and remodeling: A biological interplay regulating tissue homeostasis and diseases. Matrix Biol.

[6] Sekiguchi R, Yamada KM (2018) Basement Membranes in Development and Disease. pp 143–191 10.1016/bs.ctdb.2018.02.005 PMC6701859 29853176

[7] Hu M, Ling Z, Ren X (2022). Extracellular matrix dynamics: tracking in biological systems and their implications. J Biol Eng.

[8] Karamanos NK, Theocharis AD, Piperigkou Z et al (2021) A guide to the composition and functions of the extracellular matrix. FEBS J febs 15776. 10.1111/febs.15776 10.1111/febs.15776 33605520

[9] Pfisterer K, Shaw LE, Symmank D, Weninger W (2021) The Extracellular Matrix in Skin Inflammation and Infection. Front Cell Dev Biol 9. 10.3389/fcell.2021.682414 10.3389/fcell.2021.682414 PMC8290172 34295891

[10] Frantz C, Stewart KM, Weaver VM (2010). The extracellular matrix at a glance. J Cell Sci.

[11] Filipe EC, Chitty JL, Cox TR (2018). Charting the unexplored extracellular matrix in cancer. Int J Exp Pathol.

[12] Cox TR (2021). The matrix in cancer. Nat Rev Cancer.

[13] Neill T, Schaefer L, Iozzo RV (2016). Decorin as a multivalent therapeutic agent against cancer. Adv Drug Deliv Rev.

[14] Neill T, Iozzo RV (2022). The Role of Decorin Proteoglycan in Mitophagy. Cancers (Basel).

[15] Dauvé J, Belloy N, Rivet R (2021). Differential MMP-14 Targeting by Lumican-Derived Peptides Unraveled by In Silico Approach. Cancers (Basel).

[16] Theocharis AD, Manou D, Karamanos NK (2019). The extracellular matrix as a multitasking player in disease. FEBS J.

[17] Afratis NA, Nikitovic D, Multhaupt HAB (2017). Syndecans – key regulators of cell signaling and biological functions. FEBS J.

[18] Karamanos NK, Piperigkou Z, Theocharis AD (2018). Proteoglycan Chemical Diversity Drives Multifunctional Cell Regulation and Therapeutics. Chem Rev.

[19] Kontio J, Soñora VR, Pesola V (2022). Analysis of extracellular matrix network dynamics in cancer using the MatriNet database. Matrix Biol.

[20] Winograd-Katz SE, Fässler R, Geiger B, Legate KR (2014). The integrin adhesome: from genes and proteins to human disease. Nat Rev Mol Cell Biol.

[21] Shannon P, Markiel A, Ozier O (2003). Cytoscape: A Software Environment for Integrated Models of Biomolecular Interaction Networks. Genome Res.

[22] Sherman BT, Hao M, Qiu J (2022). DAVID: a web server for functional enrichment analysis and functional annotation of gene lists (2021 update). Nucleic Acids Res.

[23] Ogris C, Guala D, Kaduk M, Sonnhammer ELL (2018). FunCoup 4: new species, data, and visualization. Nucleic Acids Res.

[24] Fonseka P, Pathan M, Chitti SV (2021). FunRich enables enrichment analysis of OMICs datasets. J Mol Biol.

[25] Kotlyar M, Pastrello C, Malik Z, Jurisica I (2019). IID 2018 update: context-specific physical protein–protein interactions in human, model organisms and domesticated species. Nucleic Acids Res.

[26] Wong AK, Krishnan A, Yao V (2015). IMP 2.0: a multi-species functional genomics portal for integration, visualization and prediction of protein functions and networks. Nucleic Acids Res.

[27] Shao X, Taha IN, Clauser KR (2020). MatrisomeDB: the ECM-protein knowledge database. Nucleic Acids Res.

[28] Clerc O, Deniaud M, Vallet SD (2019). MatrixDB: integration of new data with a focus on glycosaminoglycan interactions. Nucleic Acids Res.

[29] Szklarczyk D, Gable AL, Nastou KC (2021). The STRING database in 2021: customizable protein–protein networks, and functional characterization of user-uploaded gene/measurement sets. Nucleic Acids Res.

[30] Izzi V, Davis MN, Naba A (2020) Pan-Cancer Analysis of the Genomic Alterations and Mutations of the Matrisome. Cancers (Basel) 12:2046. 10.3390/cancers12082046 10.3390/cancers12082046 PMC7463652 32722287

[31] Berthollier C, Vallet SD, Deniaud M et al (2021) Building Protein-Protein and Protein‐Glycosaminoglycan Interaction Networks Using MatrixDB, the Extracellular Matrix Interaction Database. 10.1002/cpz1.47. Curr Protoc 1:10.1002/cpz1.47 33794052

[32] Chastney MR, Conway JRW, Ivaska J (2021). Integrin adhesion complexes. Curr Biol.

[33] Kechagia JZ, Ivaska J, Roca-Cusachs P (2019). Integrins as biomechanical sensors of the microenvironment. Nat Rev Mol Cell Biol.

[34] Kyriakopoulou K, Kefali E, Piperigkou Z (2018). Advances in targeting epidermal growth factor receptor signaling pathway in mammary cancer. Cell Signal.

[35] Rammal H, Saby C, Magnien K et al (2016) Discoidin Domain Receptors: Potential Actors and Targets in Cancer. Front Pharmacol 7. 10.3389/fphar.2016.00055 10.3389/fphar.2016.00055 PMC4789497 27014069

[36] Chung H, Multhaupt HAB, Oh E-S, Couchman JR (2016). Minireview: Syndecans and their crucial roles during tissue regeneration. FEBS Lett.

[37] Czarnowski D (2021). Syndecans in cancer: A review of function, expression, prognostic value, and therapeutic significance. Cancer Treat Res Commun.

[38] Senbanjo LT, Chellaiah MA (2017) CD44: A Multifunctional Cell Surface Adhesion Receptor Is a Regulator of Progression and Metastasis of Cancer Cells. Front Cell Dev Biol 5. 10.3389/fcell.2017.00018 10.3389/fcell.2017.00018 PMC5339222 28326306

[39] Xu H, Niu M, Yuan X (2020). CD44 as a tumor biomarker and therapeutic target. Exp Hematol Oncol.

[40] Hassn Mesrati M, Syafruddin SE, Mohtar MA, Syahir A (2021). CD44: A Multifunctional Mediator of Cancer Progression. Biomolecules.

[41] Piperigkou Z, Kyriakopoulou K, Koutsakis C (2021). Key Matrix Remodeling Enzymes: Functions and Targeting in Cancer. Cancers (Basel).

[42] Piperigkou Z, Karamanos NK (2021). Matrix Effectors and Cancer. Cancers (Basel).

[43] Franchi M, Masola V, Bellin G (2019). Collagen Fiber Array of Peritumoral Stroma Influences Epithelial-to-Mesenchymal Transition and Invasive Potential of Mammary Cancer Cells. J Clin Med.

[44] Winkler J, Abisoye-Ogunniyan A, Metcalf KJ, Werb Z (2020). Concepts of extracellular matrix remodelling in tumour progression and metastasis. Nat Commun.

[45] Koorman T, Jansen KA, Khalil A (2022). Spatial collagen stiffening promotes collective breast cancer cell invasion by reinforcing extracellular matrix alignment. Oncogene.

[46] Kubow KE, Vukmirovic R, Zhe L (2015). Mechanical forces regulate the interactions of fibronectin and collagen I in extracellular matrix. Nat Commun.

[47] Ghasemi H, Mousavibahar SH, Hashemnia M (2021). Transitional cell carcinoma matrix stiffness regulates the osteopontin and YAP expression in recurrent patients. Mol Biol Rep.

[48] Kyriakopoulou K, Riti E, Piperigkou Z (2020). ΕGFR/ERβ-Mediated Cell Morphology and Invasion Capacity Are Associated with Matrix Culture Substrates in Breast Cancer. Cells.

[49] Kapp TG, Rechenmacher F, Neubauer S (2017). A Comprehensive Evaluation of the Activity and Selectivity Profile of Ligands for RGD-binding Integrins. Sci Rep.

[50] Franchi M, Piperigkou Z, Riti E et al (2020) Long filopodia and tunneling nanotubes define new phenotypes of breast cancer cells in 3D cultures. Matrix Biol Plus 100026. 10.1016/j.mbplus.2020.100026 10.1016/j.mbplus.2020.100026 PMC7852320 33543024

[51] Couchman JR (2021). Syndecan-1 (CD138), Carcinomas and EMT. Int J Mol Sci.

[52] Kyriakopoulou K, Kefali E, Piperigkou Z (2021). EGFR is a pivotal player of the E2/ERβ – mediated functional properties, aggressiveness, and stemness in triple-negative breast cancer cells. FEBS J.

[53] Li W, Ma H, Zhang J (2017). Unraveling the roles of CD44/CD24 and ALDH1 as cancer stem cell markers in tumorigenesis and metastasis. Sci Rep.

[54] Gzil A, Zarębska I, Bursiewicz W (2019). Markers of pancreatic cancer stem cells and their clinical and therapeutic implications. Mol Biol Rep.

[55] Sonbol H (2018). Extracellular matrix remodeling in human disease. J Microsc Ultrastruct.

[56] Ford AJ, Rajagopalan P (2018) Extracellular matrix remodeling in 3D: implications in tissue homeostasis and disease progression. WIREs Nanomed Nanobiotechnol 10. 10.1002/wnan.1503 10.1002/wnan.1503 29171177

[57] Bihlet AR, Karsdal MA, Sand JMB (2017). Biomarkers of extracellular matrix turnover are associated with emphysema and eosinophilic-bronchitis in COPD. Respir Res.

[58] Reimann C, Brangsch J, Colletini F (2017). Molecular imaging of the extracellular matrix in the context of atherosclerosis. Adv Drug Deliv Rev.

[59] Christensen G, Herum KM, Lunde IG (2019). Sweet, yet underappreciated: Proteoglycans and extracellular matrix remodeling in heart disease. Matrix Biol.

[60] Järveläinen H, Sainio A, Wight TN (2015). Pivotal role for decorin in angiogenesis. Matrix Biol.

[61] Miller RE, Ishihara S, Tran PB et al (2018) An aggrecan fragment drives osteoarthritis pain through Toll-like receptor 2. 10.1172/jci.insight.95704. JCI Insight 3:10.1172/jci.insight.95704 PMC5926921 29563338

[62] Wang Y, Li Y, Khabut A (2017). Quantitative proteomics analysis of cartilage response to mechanical injury and cytokine treatment. Matrix Biol.

[63] Pérez-García S, Carrión M, Gutiérrez-Cañas I (2019). Profile of Matrix-Remodeling Proteinases in Osteoarthritis: Impact of Fibronectin. Cells.

[64] Han B, Li Q, Wang C (2021). Differentiated activities of decorin and biglycan in the progression of post-traumatic osteoarthritis. Osteoarthr Cartil.

[65] Abyaneh HS, Regenold M, McKee TD (2020). Towards extracellular matrix normalization for improved treatment of solid tumors. Theranostics.

[66] Piperigkou Z, Tzaferi K, Makrokanis G (2022). The microRNA-cell surface proteoglycan axis in cancer progression. Am J Physiol Physiol.

[67] Pourhanifeh MH, Mohammadi R, Noruzi S (2019). The role of fibromodulin in cancer pathogenesis: implications for diagnosis and therapy. Cancer Cell Int.

[68] Ao Z, Yu S, Qian P (2017). Tumor angiogenesis of SCLC inhibited by decreased expression of FMOD via downregulating angiogenic factors of endothelial cells. Biomed Pharmacother.

[69] Gubbiotti MA, Neill T, Iozzo RV (2017). A current view of perlecan in physiology and pathology: A mosaic of functions. Matrix Biol.

[70] Bouris P, Manou D, Sopaki-Valalaki A (2018). Serglycin promotes breast cancer cell aggressiveness: Induction of epithelial to mesenchymal transition, proteolytic activity and IL-8 signaling. Matrix Biol.

[71] Sheehy EJ, Cunniffe GM, O’Brien FJ (2018) Collagen-based biomaterials for tissue regeneration and repair. Peptides and Proteins as Biomaterials for Tissue Regeneration and Repair. Elsevier, pp 127–150

[72] Passi A, Vigetti D (2019). Hyaluronan as tunable drug delivery system. Adv Drug Deliv Rev.

[73] La Noce M, Stellavato A, Vassallo V (2021). Hyaluronan-Based Gel Promotes Human Dental Pulp Stem Cells Bone Differentiation by Activating YAP/TAZ Pathway. Cells.

[74] Walimbe T, Panitch A (2020) Proteoglycans in Biomedicine: Resurgence of an Underexploited Class of ECM Molecules. Front Pharmacol 10. 10.3389/fphar.2019.01661 10.3389/fphar.2019.01661 PMC7000921 32082161

[75] Jääskeläinen A, Jukkola A, Risteli J (2019). Elevated preoperative serum levels of collagen I carboxyterminal telopeptide predict better outcome in early-stage luminal-B-like (HER2-negative) and triple-negative subtypes of breast cancer. Tumor Biol.

[76] Liu B, Zhao Y, Yuan J (2017). Elevated N-telopeptide as a potential diagnostic marker for bone metastasis in lung cancer: A meta-analysis. PLoS ONE.

[77] Thorlacius-Ussing J, Kehlet SN, Rønnow SR (2019). Non-invasive profiling of protease-specific elastin turnover in lung cancer: biomarker potential. J Cancer Res Clin Oncol.

[78] Kumavat R, Kumar V, Malhotra R (2021). Biomarkers of Joint Damage in Osteoarthritis: Current Status and Future Directions. Mediators Inflamm.

[79] Karousou E, Misra S, Ghatak S (2017). Roles and targeting of the HAS/hyaluronan/CD44 molecular system in cancer. Matrix Biol.

[80] Appunni S, Anand V, Khandelwal M (2019). Small Leucine Rich Proteoglycans (decorin, biglycan and lumican) in cancer. Clin Chim Acta.

[81] Park YS, Kim DS, Cho SW (2018). Analysis of Syndecan-2 Methylation in Bowel Lavage Fluid for the Detection of Colorectal Neoplasm. Gut Liver.

[82] Bertrand J, Bollmann M (2019). Soluble syndecans: biomarkers for diseases and therapeutic options. Br J Pharmacol.

[83] Frampton AE, Prado MM, López-Jiménez E (2018). Glypican-1 is enriched in circulating-exosomes in pancreatic cancer and correlates with tumor burden. Oncotarget.

[84] Zhou Y, Horowitz JC, Naba A (2018). Extracellular matrix in lung development, homeostasis and disease. Matrix Biol.

[85] Javdani H, Mollaei H, Karimi F (2022). Review article epithelial to mesenchymal transition–associated microRNAs in breast cancer. Mol Biol Rep.

[86] Abdel-Hamid NM, Abass SA (2021). Matrix metalloproteinase contribution in management of cancer proliferation, metastasis and drug targeting. Mol Biol Rep.

